# Biphasic Regulation of Lipid Metabolism: A Meta-Analysis of Icodextrin in Peritoneal Dialysis

**DOI:** 10.1155/2015/208980

**Published:** 2015-12-15

**Authors:** Yan-Feng Huang, Da-Jian Zhu, Xiao-Wu Chen, Man-Zhao Ouyang, Wei-Jie Zhang

**Affiliations:** Department of Gastrointestinal Surgery, Shunde First People's Hospital Affiliated to Southern Medical University, Guangdong 528300, China

## Abstract

*Objectives*. The objective of this systematic meta-analysis was to study the impact of icodextrin (ICO) on lipid profiles.* Methods*. MEDLINE, PubMed, Embase, Chinese Biomedical Literature, and the Cochrane Library and Reference lists were searched (last search September 2014) in accordance with the Cochrane Handbook for Systematic Reviews of Interventions.* Results*. Searches identified 13 eligible trials with a total of 850 patients. The differentials of total cholesterol (TC) and free fatty acid (FFA) in the ICO group were greater than those in the GLU group. Metaregression analysis showed that TC levels positively correlated with its baseline levels. In the subgroup of patients with dialysis duration more than 6 months, TC and TG in the ICO group were less. In pooled data from cross-sectional studies, differential of TG in the ICO group was less. In the subgroup of patients with diabetes (Martikainen et al., 2005, Sniderman et al., 2014, and Takatori et al., 2011), differential of high-density lipoprotein cholesterol (HDL-C) in the ICO group was less. There was no significant effect on low-density lipoprotein cholesterol (LDL-C), very low-density lipoprotein cholesterol (VLDL-C), or lipoprotein(a).* Conclusions*. ICO may be beneficial to lipid metabolism, especially for its biphasic regulation of plasma TC levels.

## 1. Introduction

Peritoneal dialysis (PD) is a widely established treatment used for renal replacement therapy [1]. It provides clinical outcomes similar to those with hemodialysis (HD) [2] and similar and possibly better quality of life [3, 4]. However, PD populations have multiple modifiable cardiovascular (CV) risk factors, including dyslipidemia, insulin resistance, hypertension, smoking, obesity, and factors that are associated with uremia (such as vascular calcification, inflammation, endothelial dysfunction, and oxidative stress) [5]. Fluid overload and glucose exposure are postulated to contribute significantly to CV mortality in PD patients [6].

Glucose is the most widely used osmotic agent for PD-based solutions. Recent studies showed it may contribute to the pathogenesis of the atherogenic dyslipoproteinemia. It has been suggested that approximately 60–80% of the glucose instilled into the peritoneal cavity is absorbed, corresponding to 100–300 g of glucose intake per day, depending on the glucose concentration and the membrane transport status of conventional glucose solutions [7]. Patients treated with PD experience an elevated exposure to metabolic risk factors, including dyslipidemia (elevated triglycerides and very low-density lipoprotein (VLDL)) and hyperglycemia, which can be aggravated by increased glucose load [8–10]. Adjusted all-cause mortality hazard ratio for time-averaged hemoglobin A1c (HbA1c) values of 7.0%–7.9% and 8.0%–8.9% was 1.10 and 1.28, respectively, compared with 6.0%–6.9% as found previously [11]. Higher HbA1c is also associated with increased CV mortality in nondiabetic patients [12].

Icodextrin (ICO) is a starch-derived, water-soluble, high-molecular-weight glucose polymer (dextrin) that is used as a colloid osmotic agent [13]. Although limiting the amount of glucose in the PD prescription has been described to improve the atherogenic lipoprotein profile in a majority of publications, some studies show conflicting results [14]. These controversies may be ascribed to inadequate data and differences in experimental designs among the published investigations. Thus far, only a limited number of studies have focused on the effects of ICO with regard to insulin resistance; however, its precise effects and mechanisms have remained unclear. A meta-analysis of published studies was conducted to clarify the sources of heterogeneity and detail the effect of the low-glucose solution on plasma lipid profiles and insulin resistance.

## 2. Methods

### 2.1. Literature Search Strategy

Comprehensive searches were carried out in MEDLINE, PubMed, Embase, Chinese Biomedical Literature (CBM), and the Cochrane Library. The searches were performed for articles published up to September 2014 relevant to lipid profile outcomes of ICO versus standard glucose (GLU) dialysate. Neither a publication year nor a publication language restriction was applied.

The search string used in PubMed was (“Peritoneal Dialysis” [Majr] AND (“Icodextrin” OR “Glucans”)) AND (“Lipids” OR “Lipid-Linked Proteins” OR “Apolipoproteins” OR “Lipid Metabolism”). Other databases were searched with comparable terms, suitable for the specific database. Reference lists of the identified relevant studies were scrutinized for additional citations.

### 2.2. Literature Screening

Studies were evaluated for inclusion by two independent researchers for relevance to the subject. A random check was performed by a supervisor. Study selection was accomplished through three phases of study screening. In phase 1, the following types of studies were excluded: reviews, case reports, letters, editorials, case-series, and papers studying nonhuman and infants. In phase 2, abstracts were reviewed for relevance and the full-text articles were obtained. In phase 3, full-text articles were reviewed; inclusion required studies describing effect of ICO versus standard GLU dialysates on lipid metabolism. Unpublished trials and conference abstracts were not included. For trials with duplicate publications, the most complete and/or more recent publication was eligible for consideration. Any discrepancies in inclusion or exclusion were resolved by discussion between the reviewers with supervision of a third person.

### 2.3. Data Extraction

Data from eligible studies were independently abstracted and summarized into a spreadsheet by two authors. Discrepancies between reviewers were resolved by consensus. The following data were extracted: country of origin, first author's surname, year of publication, type of study (randomized controlled trials (RCTs), prospective trial, or cross-sectional study), participant characteristics (sample size, mean age, sex, body mass index (BMI), type of renal replacement therapy, dialysis time, duration of intervention, reasons for withdrawal, and dropouts), serum lipid and apolipoprotein level, serum glucose level, and serum insulin level.

### 2.4. Quality Assessment

RCTs were evaluated for quality assessment in terms of randomization, allocation concealment, double-blind design, and reasons for withdrawals. Each study was scored based on the Jadad scale, in which scores ranged from 0 to 5. According to the Jadad scale, 0 or 1 point was assigned to each of the five items as follows: with or without randomization, whether the investigator used the appropriate randomization methods or not, with or without a double-blind design of experiment, whether the investigator used the appropriate double-blind design or not, and the number of withdrawals and reasons for dropouts. A Jadad score of less than 2 was classified as a low-quality study, a score of 3-4 was classified as a good-quality study, and a score of 5 was classified as an excellent-quality study. The quality assessment of the meta-analysis of RCTs is given in Table 1.

The quality of cohort studies was assessed using the Newcastle-Ottawa Scale (http://www.ohri.ca/programs/clinical_epidemiology/oxford.asp). Two authors independently assigned stars to each eligible study, taking into consideration the representativeness of the exposed cohort, ascertainment of exposure, assessment of outcome, adequacy of follow-up time for outcomes to occur, and adequacy of follow-up of cohorts.

### 2.5. Data Analysis

Statistical analyses were performed using Stata Version 10.0 (Stata Corp LP, College Station, TX, USA) software. Heterogeneity of trial results was assessed by calculating *χ* ^2^ test *P* value. Statistically significant heterogeneity was defined as *P* value less than 0.1 in *χ* ^2^ test or *I* ^2^ statistic greater than 50%; in such case a random-effects model was used. Otherwise, a fixed-effect model would have been chosen. Mean values, the standard deviation (SD), and the number of patients (*N*) were tabulated separately for the ICO solution group and the standard glucose solution (GLU) group and the weighted mean difference (WMD) and 95% confidence interval (CI) were calculated. To explore the sources of heterogeneity, a metaregression analysis was performed. Subgroup analyses were also used to evaluate the effect in various conditions. Significance was then tested by *Z*-test, and *P* values less than 0.05 were considered statistically significant.

## 3. Results

### 3.1. Study Characteristics and Quality Assessment

**Figure 1:**
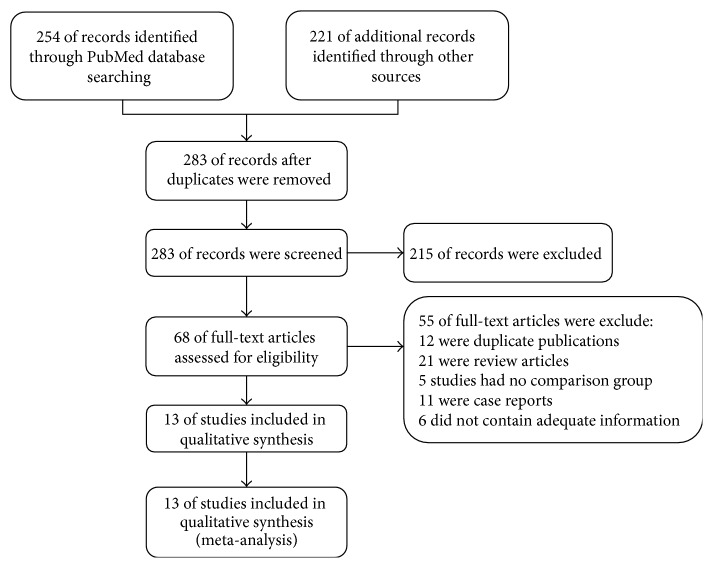
Flow chart indicating the number of citations retrieved by individual searches and the final number of included trials; reasons for exclusions are provided.

A total of 283 potentially relevant citations were identified and screened. The date of the last access was September 2014. Of those articles, 68 were assessed in full text to decide whether they fulfilled the inclusion criteria. A total of 55 articles were excluded (12 were duplicate publications, 21 were review articles, 5 studies had no comparison group, 11 were case reports, and 6 did not contain adequate information), yielding 13 studies, representing 3 prospective trials [24–26], 4 randomized control trials [14–16, 20], and 6 cross-sectional studies [17–19, 21–23] (Figure 1). The included studies were published from 2001 to 2014 and reported data from 1999 to 2011. The included trials involved 850 participants; the characteristics of these studies are summarized in Tables 1 and 2.

According to the Newcastle-Ottawa Scale, of the cohort studies, one was considered to be of fair (scale of 6) quality and two were of good (scale of 7) quality (Table 1). As for the Jadad scores for the four randomized controlled trials, three trials were given scores of 3 and one was given a score of 4 (Table 2).

### 3.2. Effect of ICO or Standard GLU Solutions on Lipid Profiles

In the crude analysis, differentials of total cholesterol (TC) and free fatty acid (FFA) in the ICO group were found to be greater than those in the GLU group after PD [16, 17, 21–26]. The pooled weighted mean differences were statistically significant (TC: *P* < 0.001; FFA: *P* = 0.004) (Table 3). The results show no difference in triglycerides (TG), HDL-C, LDL-C, VLDL-C, and lipoprotein(a) [Lip(a)] (*P* > 0.05). The differential of serum albumin (ALB) in the ICO group was less than that in the GLU group (*P* = 0.031). The results show no difference in BMI, body weight, HbA1c, or insulin (*P* > 0.05) (Supplemental Table  1 in Supplementary Material available online at http://dx.doi.org/10.1155/2015/208980).

In the subgroup of patients with dialysis duration more than 6 months [15, 16, 25], differentials of TC and TG in the ICO group were less than those in the GLU group (TC: *P* < 0.001; TG: *P* = 0.004). However, the altered levels of TC and TG were not significant in the subgroup of patients with dialysis duration less than 6 months (*P* > 0.05) (Table 5).

### 3.3. Effect of ICO or Standard GLU Solutions on Lipid Profiles of Cross-Sectional Studies

In the pooled data of cross-sectional studies [17–19], the differential of TG in the ICO group was less than that in GLU group (*P* = 0.001) (Table 4). Body weight in the ICO group was higher than that in the GLU group (*P* = 0.001). The results show no difference in BMI, continuous ambulatory peritoneal dialysis (CAPD) duration, apolipoprotein B (APO-B), ALB, TC, TG LDL-C, HDL-C, VLDL-C, and serum glucose (*P* > 0.05) (Supplemental Table  2).

### 3.4. Results of Metaregression Analysis

**Figure 2:**
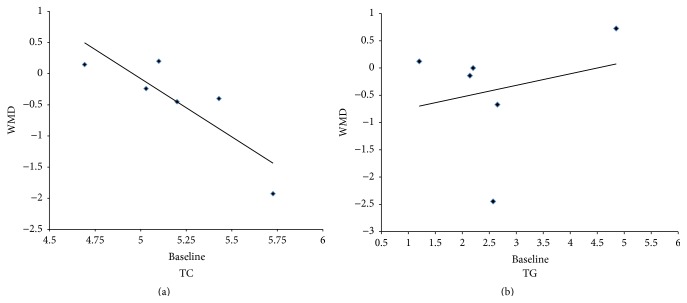
Metaregression data of TC (a) and TG (b) levels based on their baselines.

To explore the sources of heterogeneity, a metaregression analysis was performed on TC and TG levels. Intervention time and baseline levels of serum TC and TG were selected as dependent variables. As shown in Figure 2, serum TC level was found to be positively correlated with its baseline levels (*β* = −1.892, 95% CI = −3.643 to −0.141, and *P* = 0.040), while TG level was not positively correlated with its baseline levels (*β* = 0.121, 95% CI = −1.157 to 1.399, and *P* = 0.806). According to the regression, it was concluded that ICO had a positive effect on TC in which the baseline level was in the normal range. On the other hand, ICO could decrease the TC level in which the baseline was in the abnormal range.

### 3.5. Effect of ICO or Standard GLU Solutions on Lipid Profiles and Glucose Mentalism in Diabetes Patients

In the subgroup of patients with diabetes [15, 16, 26], the data showed that the differential of HDL-C in the ICO group was less than that in the GLU group (*P* = 0.002) (Table 6) and insulin levels in the ICO group were lower than those in the GLU group (*P* = 0.01) (Supplemental Table  4). In the subgroup of patients without diabetes, differentials of HbA1c and insulin in the ICO group were less than those in the GLU group (HbA1c: *P* < 0.001; insulin: *P* = 0.017) (Supplemental Table  4).

In the subgroup of patients with diabetes, the effect was also investigated according to dialysis duration. In the studies of patients with dialysis duration more than 6 months [15, 16], the differential of HDL-C level in the ICO group was less than that in the GLU group (*P* = 0.002), but the difference was not statistically significant in patients with dialysis duration less than 6 months (*P* > 0.05). In addition, TC levels in the ICO group were lower than levels in the GLU group (*P* = 0.027) (Table 7).

## 4. Discussion

PD patients suffer from a disturbed lipid metabolism, which contributes to enhanced rates of incidence of atherosclerosis and cardiovascular mortality [27]. Use of statins has been reported to result in a reduction in death from all causes and cardiac causes in CKD patients [28], and the benefit may depend on the duration of treatment [29]. Low-glucose PD was considered to reverse these abnormalities by reducing glucose delivery. However, it is still controversial whether ICO can improve the lipid metabolism of PD populations.

The meta-analysis showed that ICO has significant advantages over the conventional glucose-based dialysate in terms of lipid profiles. According to our study, ICO decreased plasma total cholesterol better than the conventional glucose solution, which are the same results as those reported by Bredie et al. [24], Hiramatsu et al. [25], and Sniderman et al. [15]. On the other hand, the study by Amici and colleagues [21], in which the TC baseline level was normal (4.69 mmol/L), showed an increase in TC with ICO treatment. To determine the cause of this discrepancy, a metaregression analysis was conducted. Interestingly, metaregression analysis indicated that the beneficial effect of ICO on TC levels was negatively related to baseline levels. Plasma TC at normal baseline levels (<5.17 mmol/L, such as in the study of Takatori et al. [16]) could be increased to a suitable level, while the level of TC could be lowered in PD populations with hypercholesterolemia. It had been reported that the effect of uremia and/or the dialysis procedure alters the effect of cholesterol level on clinical outcomes [30]. Thus, greater cholesterol levels within the normal range are associated with lower mortality risk. The biphasic regulation of ICO could benefit the balance of plasma TC levels and lead to an improvement in cardiovascular outcomes.

Cross-sectional studies showed that ICO can decrease TG levels. Another notable finding was that the improvement in TG levels and increase in the concentrations of HDL-C were better in a long-term intervention with ICO (≥6 months; *P* = 0.004 and *P* = 0.002, resp.). According to the results, therefore, ICO administration seemed to improve both TG and HDL-C levels in a time-dependent manner. Another unexpected result observed in the present study was the fact that FFA levels were higher in the ICO group than in the GLU group. Considering that measurements were performed only in a relatively small number of samples (2 studies, *n* = 61), further studies are required to provide stronger support for this conclusion.

Dyslipidemia and decreased HDL-C, as well as hypertension, are implicated in the increased CV risk in CKD patients. Di Angelantonio et al. [31] suggested that statin therapy had beneficial effects on dialysis patients, although there was a trend to be less effective with longer duration of therapy. Statin therapy also helped in reducing risk of CV mortality and deaths from all causes [32]. Taking the predictor effect of TC, TG, and HDL-C for atherosclerotic cardiovascular diseases (ASCVDs) into consideration [31], we suggest that PD populations could get greater benefit from ICO treatment by long-time intervention. However, it was unclear whether patients in the 13 studies received any lipid-lowering medications during the study period or whether their lipid-lowering medication dose remained unchanged. Further prospective studies are required to strengthen support for the conclusion.

## 5. Limitations

The potential limitation of this meta-analysis is the fact that not all trials were randomized. In addition, most trials were of short duration and had small subject numbers, which could limit assessment of long-term effects of ICO versus standard glucose solutions on lipid metabolism. Unpublished trials were not included in this study; hence, publication bias may be a limitation to the findings of this study.

## 6. Conclusions

ICO treatment may improve lipid metabolism by its biphasic regulation of plasma TC levels. Moreover, ICO may improve TG and HDL-C metabolism in a time-dependent manner, with diminished transfer of glucose from the dialysis solution being the potential mechanism. Further studies are warranted in PD patients who are undergoing treatment with hypolipidemic drugs to properly evaluate the effects of ICO on lipid profiles.

## Supplementary Material

Table 1, 2, 3, 4, 5 represented the results of meta-analysis effect of icodextrin on glucose mentalism. Figure 1 to figure 35 are forest plots of comparison between icodextrin and glucose groups on lipid profiles.

## Figures and Tables

**Table 1:** Characteristics of RCTs and cross-sectional studies included in the meta-analysis.

Study	Year	Country	Study type	Sample size	Modality	Intervention duration (months)	Solution type	Number	Mean age (yr)^a^	Gender (M/F)^b^	Study quality (Jadad)
Sniderman et al. [15]	2014	Canada	RCT	251	CAPD/APD	* *6	ICO	124	57 ± 12	64/60	4
							GLU	127	58 ± 13	59/68	

Takatori et al. [16]	2011	Japan	RCT	41	N.A.	* *24	ICO	21	55.90 ± 11.16	14/7	3
							GLU	20	56.50 ± 9.86	13/7	

Kanda et al. [17]	2013	Japan	Cross-sectional	49	CAPD/APD	* *N.A.	ICO	26	63.5 ± 11.4	19/7	N.A.
							GLU	23	64.9 ± 12.5	19/4	

Kanbay et al. [18]	2007	Turkey	Cross-sectional	67	CAPD	* *≥6	ICO	31	49 ± 16.1	17/14	N.A.
							GLU	18	40.8 ± 16.2	12/6	

Canbakan and Şahin [19]	2007	Turkey	Cross-sectional	44	CAPD	* *36.2 ± 23.7	ICO	17	49.24 ± 13.25	10/7	N.A.
							GLU	27	46.81 ± 16.28	11/16	

Lin et al. [20]	2009	China	RCT	201	CAPD	* *1	ICO	98	56.8 ± 13.5	51/47	3
							GLU	103	55.4 ± 14.0	45/58	

Paniagua et al. [14]	2009	México	RCT	59	N.A.	* *12	ICO	30	58.9 ± 7.9	12/18	3
							GLU	29	60.5 ± 9.3	16/13	

Amici et al. [21]	2001	Italy	Cross-sectional	27	CAPD	* *17	ICO	15	64 ± 16	N.A.	N.A.
							GLU	12	60 ± 16	N.A.	

Furuya et al. [22]	2006	Japan	Cross-sectional	24	CAPD/CCPD	* *6	ICO	12	57.17 ± 8.68	5/7	N.A.
							GLU	12	57.67 ± 9.18	5/7	

van Hoeck et al. [23]	2003	Belgium	Cross-sectional	16	NIPD	* *0.25	ICO	8	5.75 ± 4.03	N.A.	N.A.
							GLU	8	5.75 ± 4.03	N.A.	

Note: RCT: randomized controlled trial; APD: automated peritoneal dialysis; CAPD: continuous ambulatory peritoneal dialysis; NIPD: nocturnal intermittent peritoneal dialysis; N.A.: not applicable; ICO: icodextrin solution; GLU: standard glucose solution. ^a^Age appears as mean, mean ± standard deviation. ^b^Sex ratio: M = male, F = female.

**Table 2:** Characteristics of cohort studies included in the meta-analysis.

Study	Year	Country	Study type	Sample size	Modality	Gender (M/F)^a^	Mean age (yr)^b^	Duration (months)	Solution type (case/control)	Number (case/control)	Quality (NOS)
Bredie et al. [24]	2001	Netherlands	Prospective cohort	21	CAPD	15/6	50.3 ± 11.8	1.5	ICO/GLU	1110	7
Hiramatsu et al. [25]	2007	Japan	Prospective cohort	28	N.A.	N.A.	54.9	12	ICO/GLU	1414	6
Martikainen et al. [26]	2005	Finland	Prospective cohort	22	CAPD	18/4	60.7 ± 2.3	2	ICO/GLU	2020	7

Note: NOS: Newcastle-Ottawa Scale; CAPD: continuous ambulatory peritoneal dialysis; N.A.: not applicable; ICO: icodextrin solution; GLU: standard glucose solution. ^a^Sex ratio: M = male, F = female. ^b^Age appears as mean, mean ± standard deviation.

**Table 3:** Effect of icodextrin use on lipid profiles (RCT and cohort studies).

Factor	Number of studies	Heterogeneity test				Weighted mean difference		
		*Q*	*I* ^2^	*P* value	Model of meta^a^	Mean	[95% CI]	*P* value
Total cholesterol (mmol/L)	7	3.70	46.10%	0.190	F	−0.292	[−432, −0.153]	<0.001^*∗*^
Triglycerides (mmol/L)	6	21.02	76.20%	0.001	R	−0.357	[−0.911,0.196]	0.206
HDL-C (mmol/L)	4	7.41	59.50%	0.06	R	0.059	[−0.041,0.159]	0.245
LDL-C (mmol/L)	4	9.87	69.60%	0.02	R	−0.017	[−0.359,0.325]	0.922
VLDL-C (mmol/L)	2	0.09	<0.01%	0.762	F	−0.125	[−0.514,0.264]	0.529
Free fatty acid (mol/L)	2	0.04	<0.01%	0.845	F	−0.031	[−0.052, −0.010]	0.004^*∗*^
Lipoprotein(a) (mg/dL)	2	8.69	88.50%	0.003	R	−14.394	[−54.401,25.613]	0.481

Note: ^**∗**^the *P* value was less than 0.05, and the WMD was considered statistically significant.

^a^Model of meta: F: Fixed, R: Random.

**Table 4:** Effect of icodextrin use on lipid profiles (cross-sectional studies).

Factor	Number of studies	Heterogeneity test				Weighted mean difference		
		*Q*	*I* ^2^	*P* value	Model of meta^a^	Mean	[95% CI]	*P* value
APO-B (mg/dL)	2	2.45	59.30%	0.117	R	−1.823	[−21.328,17.682]	0.855
Total cholesterol (mmol/L)	6	1.17	<0.01%	0.947	F	−0.527	[−13.545,12.491]	0.937
Triglycerides (mmol/L)	5	4.68	14.5%	0.322	F	−34.884	[−55.617, −14.152]	0.001^*∗*^
HDL-C (mmol/L)	4	9.27	67.60%	0.026	R	4.216	[−2.560,10.991]	0.223
LDL-C (mmol/L)	2	0.09	<0.01%	0.77	F	13.784	[−2.450,30.017]	0.096
VLDL-C (mmol/L)	2	0.01	<0.01%	0.916	F	0.363	[−6.268,6.993]	0.915

Note: ^*∗*^the *P* value was less than 0.05, and the WMD was considered statistically significant.

^a^Model of meta: F: Fixed, R: Random.

**Table 5:** Effect of icodextrin on lipid profiles (in subgroups of duration).

Factor	Duration	Number of studies	Heterogeneity test				Weighted mean difference		
			*Q*	*I* ^2^	*P* value	Model of meta^a^	Mean	[95% CI]	*P* value
Total cholesterol (mmol/L)	<6 month	6	9.49	47.30%	0.091	F	−0.124	[−0.269,0.022]	0.096
	≥6 month	4	8.50	43.40%	0.112	F	−0.948	[−1.211, −0.686]	<0.001^*∗*^

Triglycerides (mmol/L)	<6 month	5	26.45	84.90%	<0.001	R	−0.456	[−0.992,0.080]	0.096
	≥6 month	3	4.32	33.6%	0.213	F	−0.602	[−1.011, −0.192]	0.004^*∗*^

HDL-C (mmol/L)	<6 month	4	1.19	<0.01%	0.754	F	−0.008	[−0.058,0.042]	0.760
	≥6 month	2	0.76	<0.01%	0.383	F	−0.033	[−0.122,0.056]	0.469

LDL-C (mmol/L)	<6 month	4	4.71	36.3%	0.194	F	−0.055	[−0.195,0.084]	0.435
	≥6 month	2	<0.01	<0.01%	0.982	F	0.098	[−0.183,0.379]	0.495

VLDL-C (mmol/L)	<6 month	4	0.09	<0.01%	0.761	F	−1.424	[−0.510,0.261]	0.527

Free fatty acid (mol/l)	<6 month	2	1.13	11.50%	0.288	F	−0.028	[−0.049, −0.007]	0.009^*∗*^

Note: ^*∗*^the *P* value was less than 0.05, and the WMD was considered statistically significant.

^a^Model of meta: F: Fixed, R: Random.

**Table 6:** Effect of icodextrin on lipid profiles (in subgroups of diabetes).

Factor	Diabetes	Number of studies	Heterogeneity test				Weighted mean difference		
			*Q*	*I* ^2^	*P* value	Model of meta	WMD	[95% CI]	*P* value
Total cholesterol (mmol/L)	Diabetes	2	2.05	51.20%	0.152	F	−0.306	[−0.655,0.044]	0.087
Triglycerides (mmol/L)	Diabetes	2	0.49	<0.01%	0.483	F	0.171	[−0.291,0.634]	0.467
HDL-C (mmol/L)	Diabetes	2	0.09	<0.01%	0.759	F	0.144	[0.055,0.233]	0.002^*∗*^
LDL-C (mmol/L)	Diabetes	2	1.45	31.00%	0.228	F	−0.197	[−0.478,0.085]	0.228

Note: ^*∗*^the *P* value was less than 0.05, and the WMD was considered statistically significant.

**Table 7:** Effect of icodextrin on lipid profiles in diabetes patients (in subgroups of duration).

Factor	Duration	Number of studies	Heterogeneity test				Weighted mean difference		
			*Q*	*I* ^2^	*P* value	Model of meta^a^	Mean	[95% CI]	*P* value
Total cholesterol (mmol/L)	<6 month	2	1.06	5.60%	0.303	F	−0.399	[−0.751, −0.046]	0.027^*∗*^
	≥6 month	2	2.05	51.20%	0.152	F	−0.306	[−0.655,0.044]	0.087

Triglycerides (mmol/L)	<6 month	2	1.63	38.60%	0.202	F	0.198	[−0.136,0.532]	0.244
	≥6 month	2	0.49	<0.01%	0.483	F	0.171	[−0.291,0.634]	0.467

HDL-C (mmol/L)	<6 month	2	0.17	<0.01%	0.677	F	0.009	[−0.082,0.100]	0.845
	≥6 month	2	0.09	<0.01%	0.759	F	0.144	[0.055,0.233]	0.002^*∗*^

LDL-C (mmol/L)	<6 month	2	2.23	55.10%	0.135	F	−0.278	[−0.565,0.008]	0.057
	≥6 month	2	1.45	31.00%	0.228	F	−0.197	[−0.478,0.085]	0.171

Note: ^*∗*^the *P* value was less than 0.05, and the WMD was considered statistically significant.

^a^Model of meta: F: Fixed, R: Random.

## Abbreviations

APO-B: Apolipoprotein B
ASCVDs: Atherosclerotic cardiovascular diseases
BMI: Body mass index
CAPD: Continuous ambulatory peritoneal dialysis
CBM: Chinese Biomedical Literature
CI: Confidence interval
CV: Cardiovascular
FFA: Free fatty acid
GLU: Glucose solution group
HD: Hemodialysis
HDL-C: High-density lipoprotein cholesterol
ICO: Icodextrin solution group
LDL-C: Low-density lipoprotein cholesterol
PD: Peritoneal dialysis
RCTs: Randomized controlled trials
TC: Total cholesterol
TG: Triglycerides
VLDL-C: Very low-density lipoprotein cholesterol
WMD: Weighted mean difference.
