# Supplementary material for: Biphasic Regulation of Lipid Metabolism: A Meta-Analysis of Icodextrin in Peritoneal Dialysis
Source: Biomed Res Int. 2015 Dec 15;2015:208980. doi: 10.1155/2015/208980 (PMC4692973; doi:10.1155/2015/208980)
Supplement: Supplementary file 1 — Table 1, 2, 3, 4, 5 represented the results of meta-analysis effect of icodextrin on glucose mentalism. Figure 1 to figure 35 are forest plots of comparison between icodextrin and glucose groups on lipid profiles. [file 208980.f1.pdf]

Table 1 Effect of icodextrin use on glucose mentalism (RCT and cohort studies).

Factor	No. of studies	Heterogeneity test				Weighted mean difference		
		Q	I^2	P-**Value**	Model of meta[a]	Mean	[95% CI]	P-**Value**
**Body mass index(kg/m2)**	2	<0.1	<0.01%	>0.999	F	-0.05	[-0.339, 0.239]	0.735
**Body weight (kg)**	3	3.45	42.00%	0.178	F	-1.806	[-5.071, 1.459]	0.278
**Albumin(g/L)**	2	0.88	0.00%	0.347	F	-0.56	[-1.068, -0.052]	0.031*
**HbA1c(%)**	4	57.16	94.80%	<0.001	R	-0.747	[-1.693, 0.199]	0.122
**Insulin (mU/L)**	2	1.42	29.60%	0.233	F	-2.768	[-11.182, 5.647]	0.519

Note: ***** The P-value was less than 0.05, and the WMD was considered statistically significant.

Table 2 Effect of icodextrin use on glucose mentalism (cross-sectional).

Factor	No. of studies	Heterogeneity test				Weighted mean difference		
		Q	I^2	P-**Value**	Model of meta[a]	Mean	[95% CI]	P-**Value**
**Body mass index(kg/m2)**	4	3.36	10.7%	0.340	F	0.433	[-0.713, 1.580]	0.459
**Body weight (kg)**	2	0.40	<0.01%	0.527	F	7.852	[3.203, 12.500]	0.001*
**CAPD duration(months)**	2	2.68	62.60%	0.102	F	3.199	[-3.829, 10.227]	0.372
**Albumin(g/L)**	3	3.10	35.40%	0.213	F	-0.160	[-0.321, 0.000]	0.050
**Glucose (mmol/L)**	3	2.27	12.0%	0.321	F	3.868	[-7.947, 15.682]	0.521
**Insulin(mU/L)**	2	0.20	<0.01%	0.659	F	-11.751	[-17.652, -5.849]	<0.001*

Note: * The *P*-value was less than 0.05, and the WMD was considered statistically significant.

Table 3 **Effect of icodextrin on glucose mentalism (in subgroups of duration).**

Factor	Duration	No. of studies	Heterogeneity test				Weighted mean difference		
			Q	I2	P-Value	Model of metaa	Mean	[95% CI]	P-Value
**Body mass index(kg/m2)**	<6 month	2	<0.01	<0.01%	>0.99	F	-0.050	[-0.339, 0.239]	0.735
**Body weight (kg)**	<6 month	3	0.49	41.60%	0.691	F	-0.655	[-3.883, 2.573]	0.454
	≥6 month	2	2.25	55.5%	0.134	F	-1.738	[-5.233, 1.756]	0.330
**Albumin(g/L)**	<6 month	2	0.24	<0.01%	0.622	F	-0.394	[-0.905, 0.117]	0.131
**Insulin (mU/L)**	<6 month	3	11.00	81.80%	0.004	R	-0.740	[-1.551, 0.070]	0.073
**HbA1c(%)**	<6 month	2	0.9	0.00%	0.342	F	-0.048	[-0.335, 0.239]	0.744

Note: * The *P*-value was less than 0.05, and the WMD was considered statistically significant.

Table 4 **Effect of icodextrin on glucose mentalism (in subgroups of diabetes).**

Factor	Diabetes	No. of studies	Heterogeneity test				Weighted mean difference		
			Q	I2	P-Value	Model of meta	WMD	[95% CI]	P-Value
**Body weight (kg)**	Diabetes	2	3.27	69.40%	0.071	R	-2.076	[-5.571, 1.419]	0.244
**Glucose (mmol/L)**	Non	2	0.86	0.00%	0.354	F	-0.175	[-0.397, 0.046]	0.12
**HbA1c(%)**	Diabetes	2	0.91	0.00%	0.343	R	-0.51	[-0.415, -0.605]	0.049*
	Non	2	1.52	33.10%	0.245	R	-0.268	[-0.418, -0.118]	<0.001*
**Insulin (mU/L)**	Diabetes	2	0.68	0.00%	0.408	R	-3.205	[-5.641, -0.769]	0.01*
	Non	2	0.22	0.00%	0.643	R	-0.791	[-1.440, -0.143]	0.017*

Note: * The *P*-value was less than 0.05, and the WMD was considered statistically significant.

Table 5 **Effect of icodextrin on glucose mentalism in diabetes patients (in subgroups of duration).**

**Factor(D)**	**Duration**	**No. of studies**	**Heterogeneity test**				**Weighted mean difference**		
			Q	I2	P-Value	Model of metaa	Mean	[95% CI]	P-Value
**Body weight (kg)**	<6 month	2	3.34	70.00%	0.068	R	-1.414	[-4.863, 2.035]	0.422
	≥6 month	2	3.27	69.40%	0.071	F	-2.076	[-5.571, 1.419]	0.244
**HbA1c(%)**	<6 month	2	1.62	38.40%	0.203	F	-0.086	[-0.619, 0.448]	0.752
**Insulin (mU/L)**	<6 month	2	5.43	81.60%	0.02	R	-3.721	[-6.130, -1.313]	0.002*

Note: * The *P*-value was less than 0.05, and the WMD was considered statistically significant.

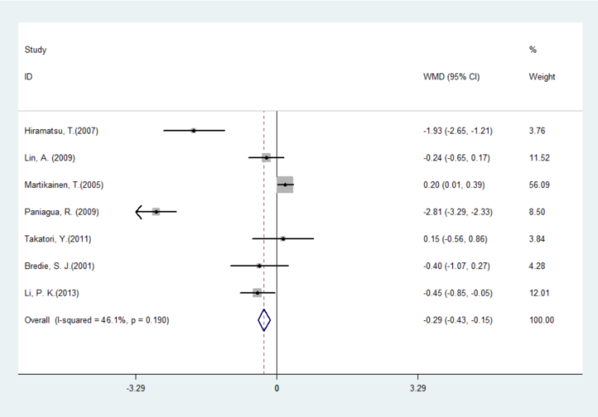

Figure 1. Forest plot of comparison (RCT and cohort studies): ICO vs GLU: Outcome: Total cholesterol (mmol/L).

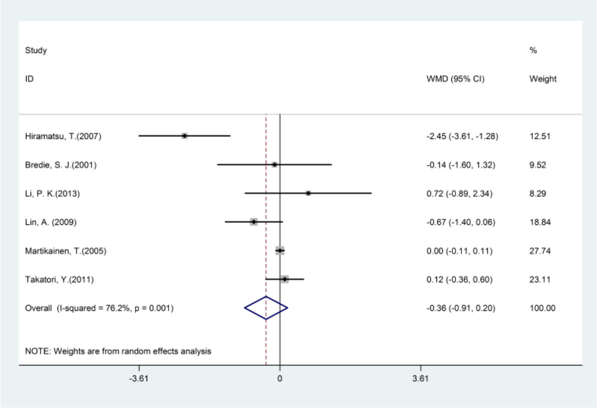

Figure 2. Forest plot of comparison (RCT and cohort studies): ICO vs GLU: Outcome: Triglycerides (mmol/L).

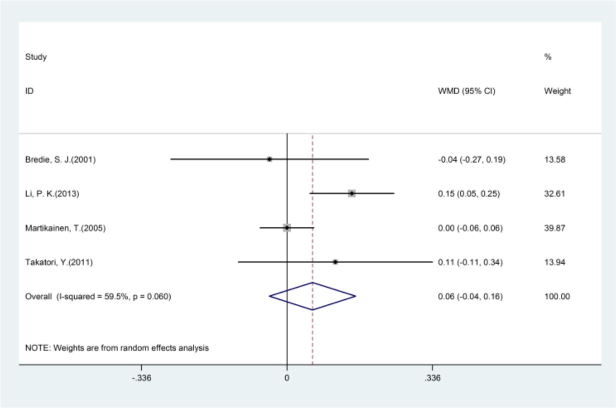

Figure 3. Forest plot of comparison (RCT and cohort studies): ICO vs GLU: Outcome: HDL-C (mmol/L).

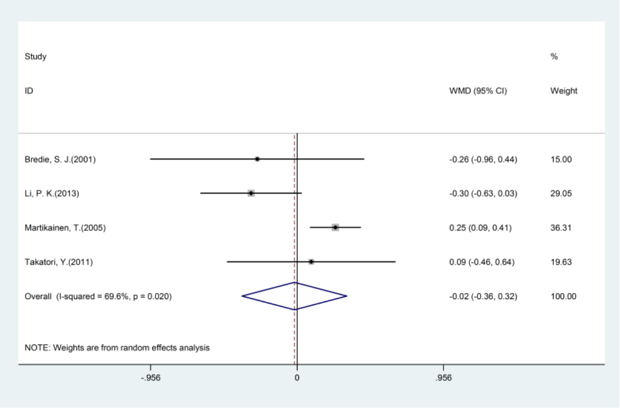

Figure 4. Forest plot of comparison (RCT and cohort studies): ICO vs GLU: Outcome: LDL-C (mmol/L).

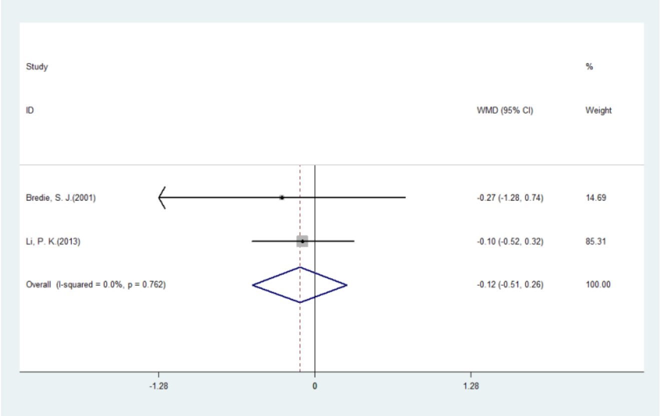

Figure 5. Forest plot of comparison (RCT and cohort studies): ICO vs GLU: Outcome: LDL-C (mmol/L).

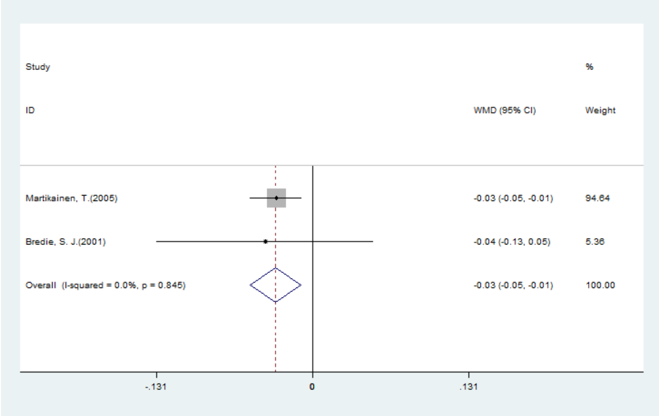

Figure 6. Forest plot of comparison (RCT and cohort studies): ICO vs GLU: Outcome: FFA (mol/L).

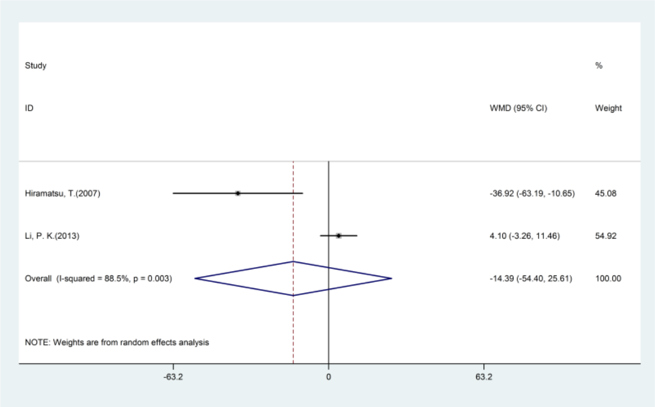

Figure 7. Forest plot of comparison (RCT and cohort studies): ICO vs GLU: Outcome: Lipoprotein (a) (mg/dL).

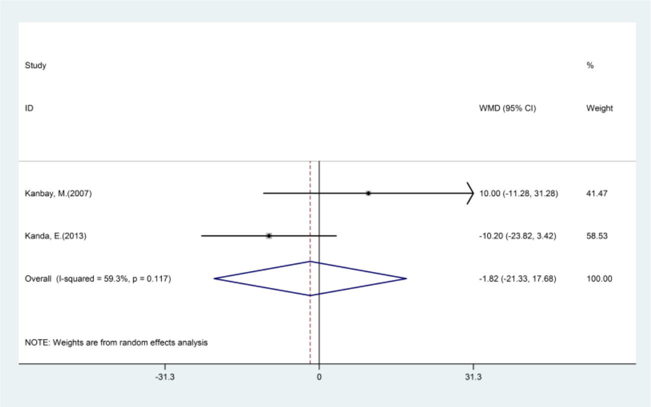

Figure 8. Forest plot of comparison (cross-sectional studies): ICO vs GLU: Outcome: APO-B (mg/dL).

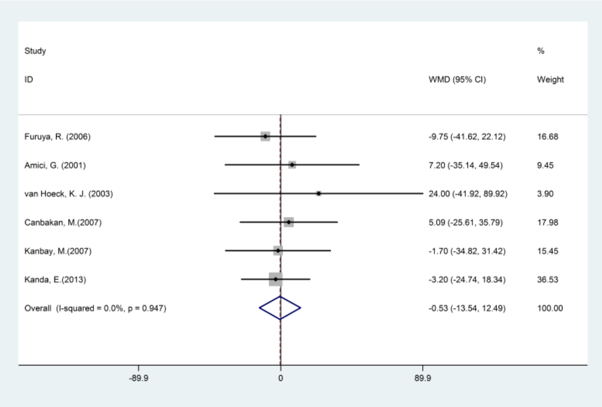

Figure 9. Forest plot of comparison (cross-sectional studies): ICO vs GLU: Outcome: Total cholesterol (mmol/L).

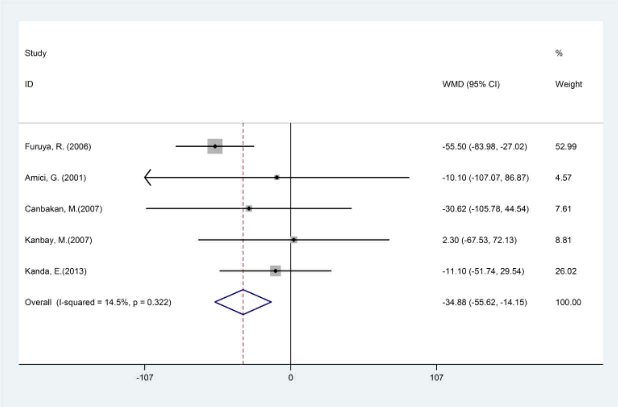

Figure 10. Forest plot of comparison (cross-sectional studies): ICO vs GLU: Outcome: Triglycerides (mmol/L).

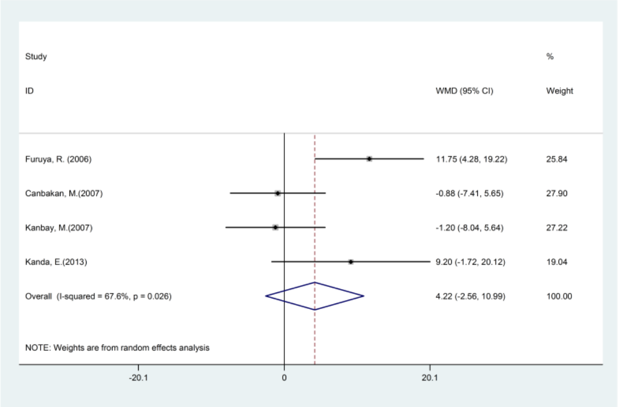

Figure 11. Forest plot of comparison (cross-sectional studies): ICO vs GLU: Outcome: HDL-C (mmol/L).

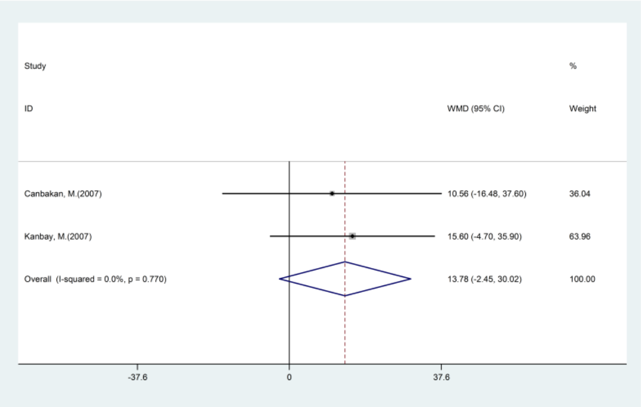

Figure 12. Forest plot of comparison (cross-sectional studies): ICO vs GLU: Outcome: LDL-C (mmol/L).

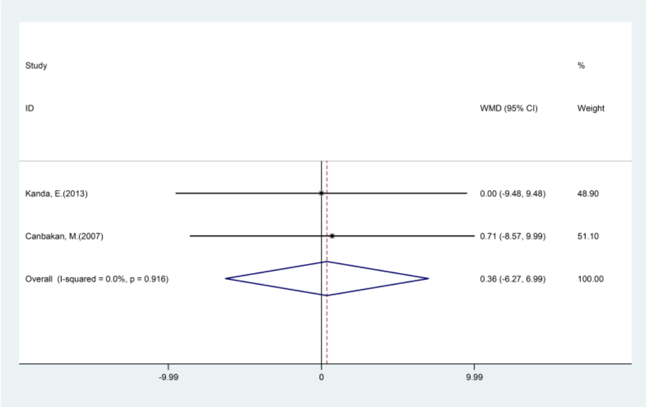

Figure 13. Forest plot of comparison (cross-sectional studies): ICO vs GLU: Outcome: VLDL-C (mmol/L).

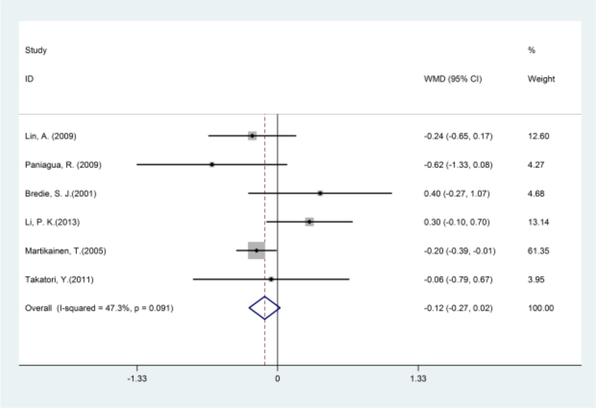

Figure 14. Forest plot of comparison (in subgroups of duration, <6 month): ICO vs GLU: Outcome: Total cholesterol (mmol/L).

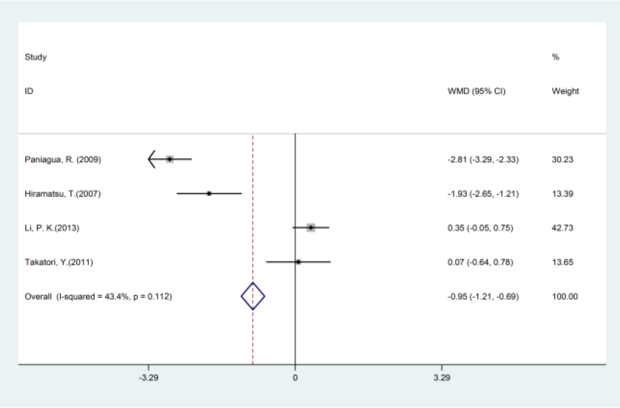

Figure 15. Forest plot of comparison (in subgroups of duration, ≥6 month): ICO vs GLU: Outcome: Total cholesterol (mmol/L).

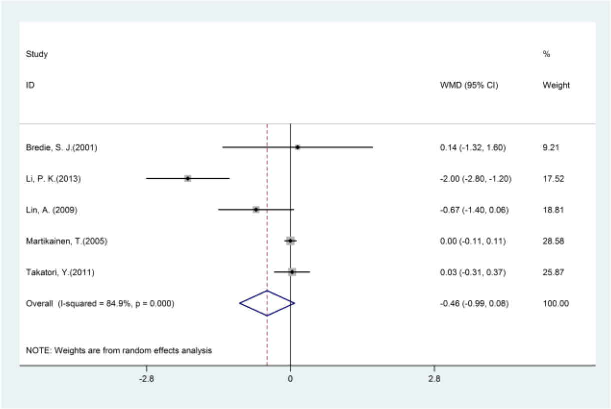

Figure 16. Forest plot of comparison (in subgroups of duration, <6 month): ICO vs GLU: Outcome: Triglycerides (mmol/L).

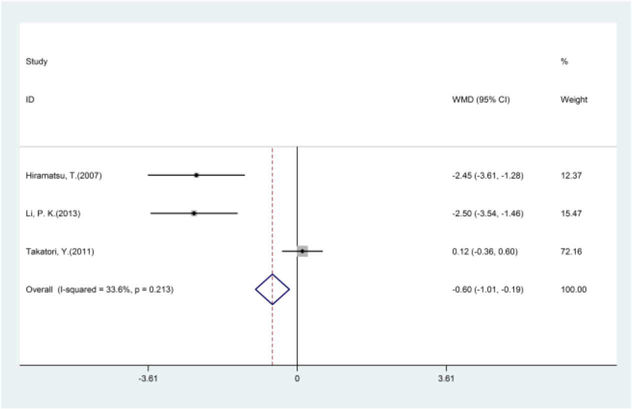

Figure 17. Forest plot of comparison (in subgroups of duration, ≥6 month): ICO vs GLU: Outcome: Triglycerides (mmol/L).

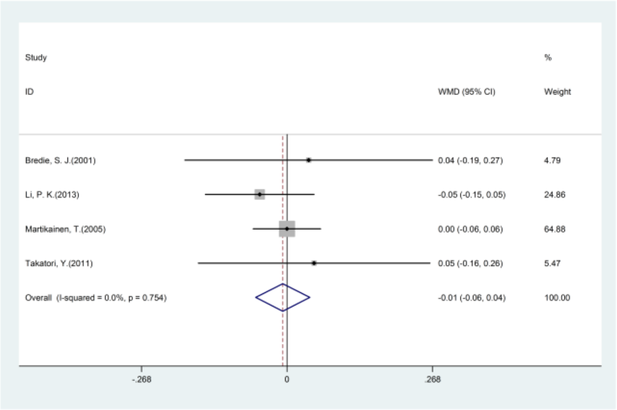

Figure 18. Forest plot of comparison (in subgroups of duration, <6 month): ICO vs GLU: Outcome: HDL-C (mmol/L).

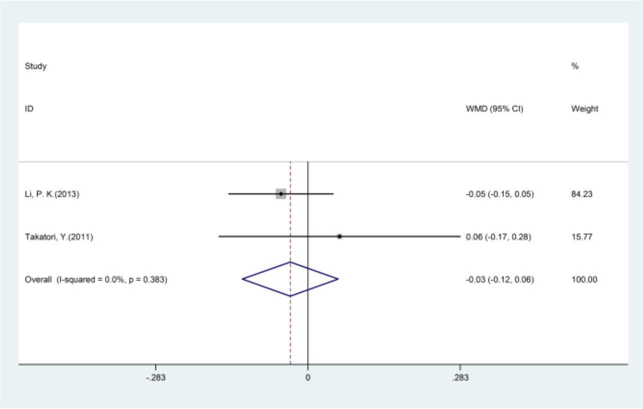

Figure 19. Forest plot of comparison (in subgroups of duration, ≥6 month): ICO vs GLU: HDL-C (mmol/L).

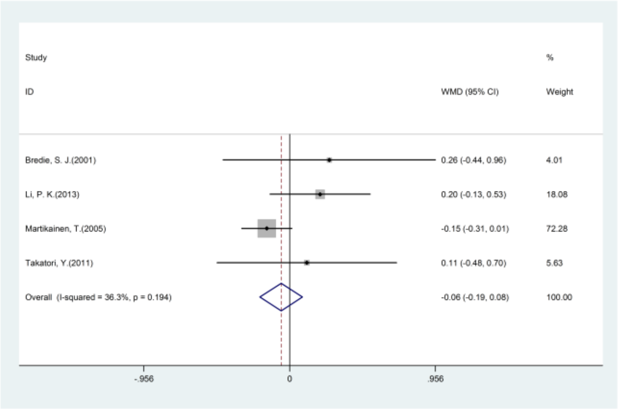

Figure 20. Forest plot of comparison (in subgroups of duration, <6 month): ICO vs GLU: Outcome: LDL-C (mmol/L).

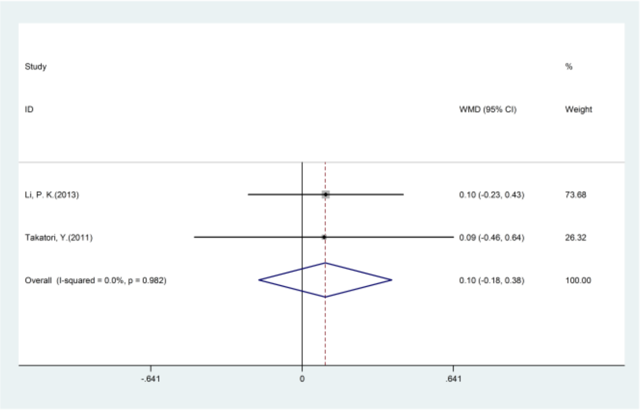

Figure 21. Forest plot of comparison (in subgroups of duration, ≥6 month): ICO vs GLU: LDL-C (mmol/L).

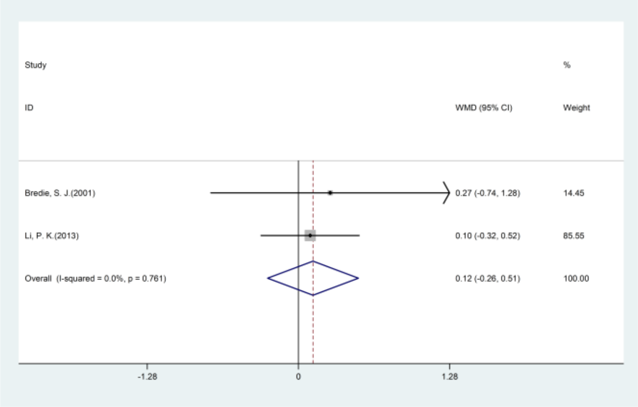

Figure 22. Forest plot of comparison (in subgroups of duration, <6 month): ICO vs GLU: Outcome: VLDL-C (mmol/L).

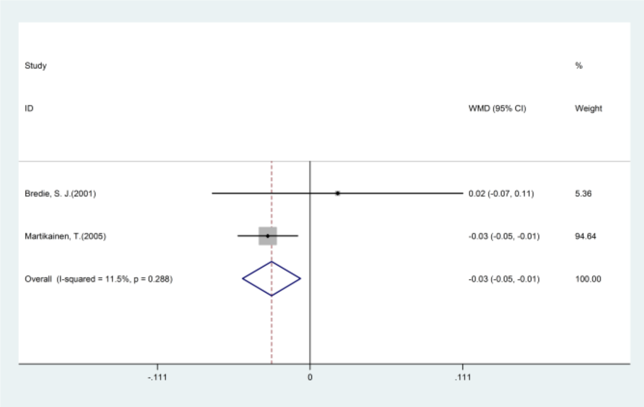

Figure 23. Forest plot of comparison (in subgroups of duration, <6 month): ICO vs GLU: Outcome: FFA (mmol/L).

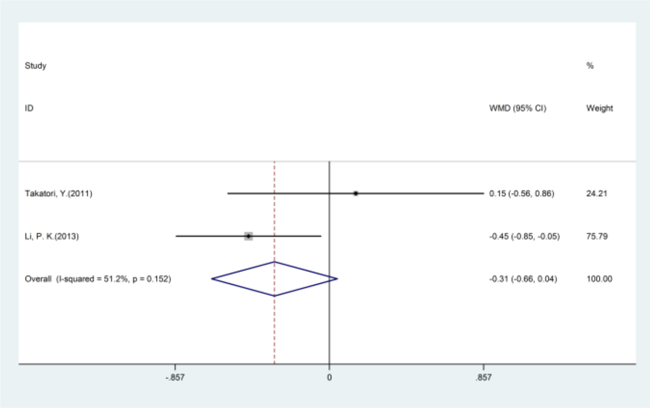

Figure 24. Forest plot of comparison (in subgroups of diabetes): ICO vs GLU: Outcome: Total cholesterol (mmol/L).

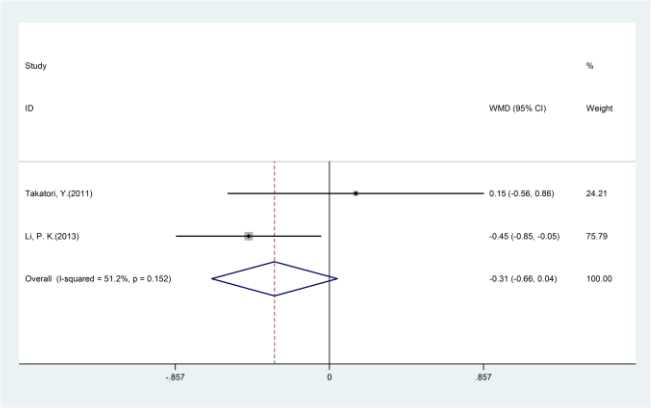

Figure 25. Forest plot of comparison (in subgroups of diabetes): ICO vs GLU: Outcome: Triglycerides (mmol/L).

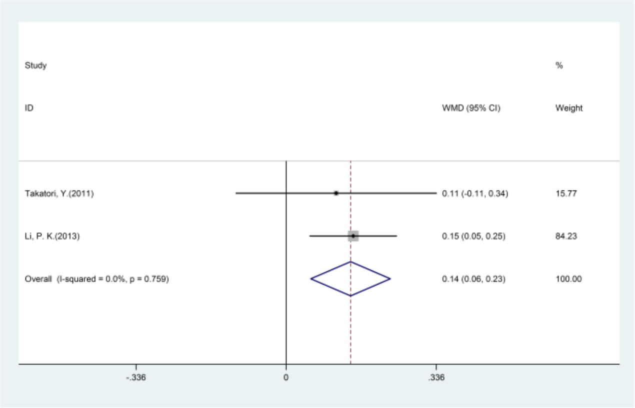

Figure 26. Forest plot of comparison (in subgroups of diabetes): ICO vs GLU: Outcome: HDL-C (mmol/L).

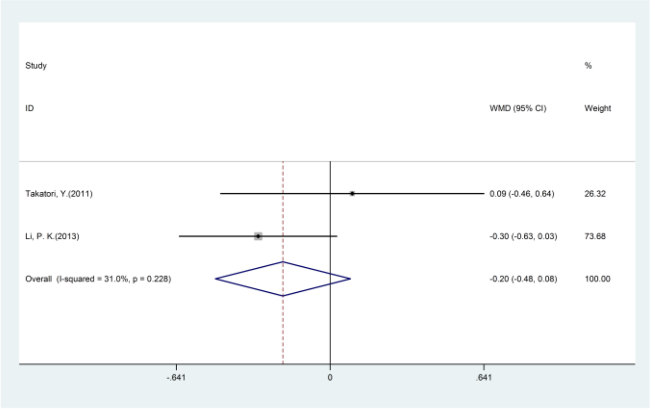

Figure 27. Forest plot of comparison (in subgroups of diabetes): ICO vs GLU: Outcome: LDL-C (mmol/L).

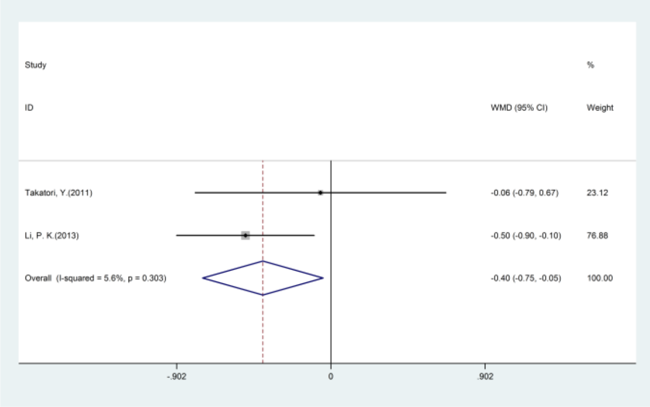

Figure 28. Forest plot of comparison (in subgroups of diabetes, <6 month): ICO vs GLU: Outcome: Total Cholesterol (mmol/L).

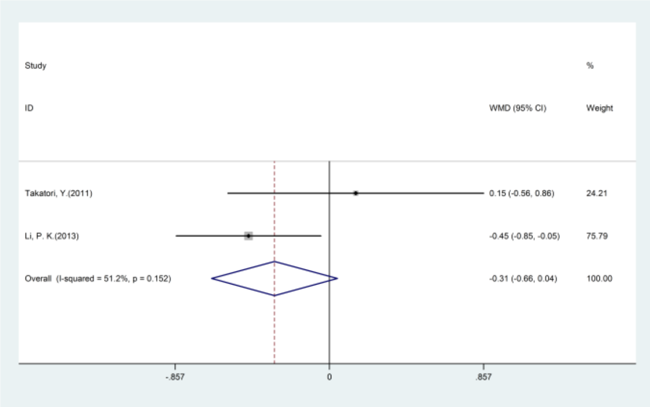

Figure 29. Forest plot of comparison (in subgroups of diabetes, ≥6 month): ICO vs GLU: Outcome: Total Cholesterol (mmol/L).

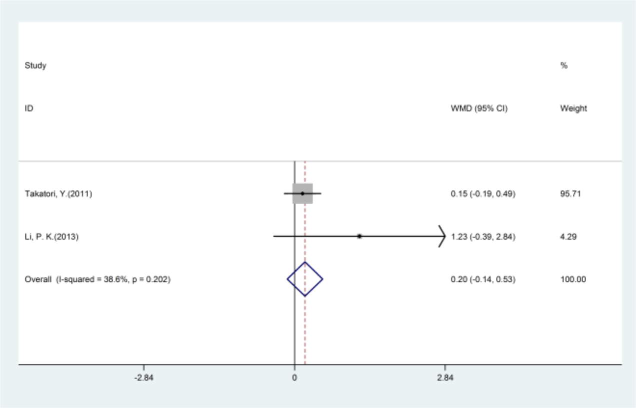

Figure 30. Forest plot of comparison (in subgroups of diabetes, <6 month): ICO vs GLU: Outcome: Triglycerides (mmol/L).

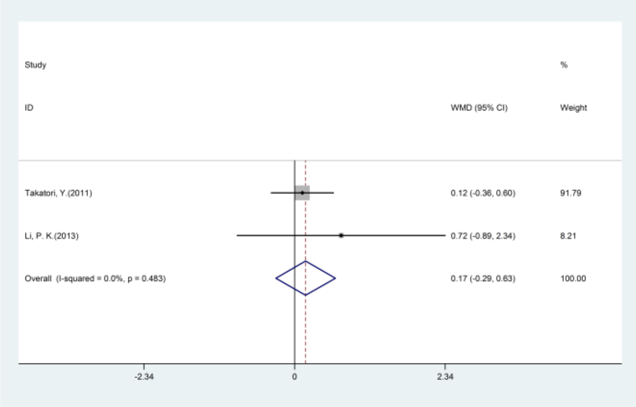

Figure 31. Forest plot of comparison (in subgroups of diabetes, ≥6 month): ICO vs GLU: Outcome: Triglycerides (mmol/L).

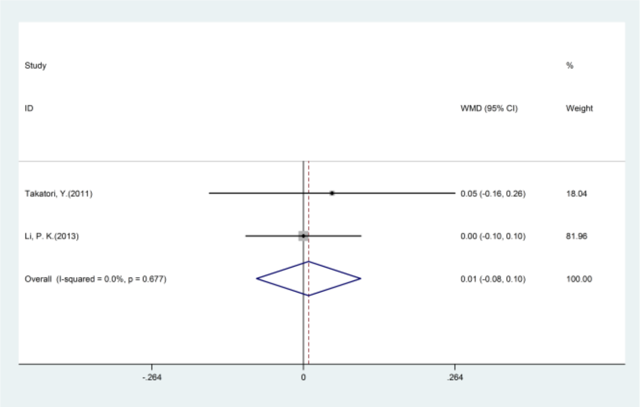

Figure 32. Forest plot of comparison (in subgroups of diabetes, <6 month): ICO vs GLU: Outcome: HDL-C (mmol/L).

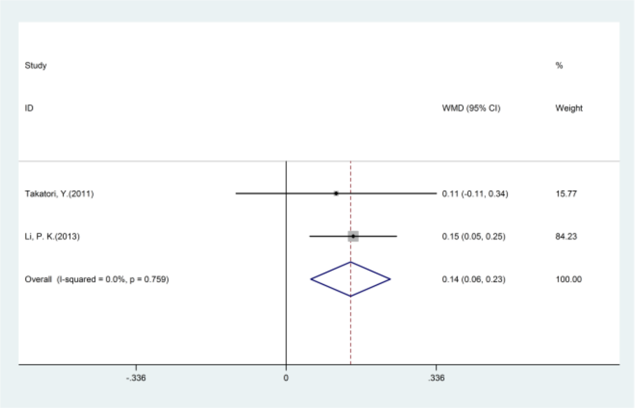

Figure 33. Forest plot of comparison (in subgroups of diabetes, ≥6 month): ICO vs GLU: Outcome: HDL-C (mmol/L).

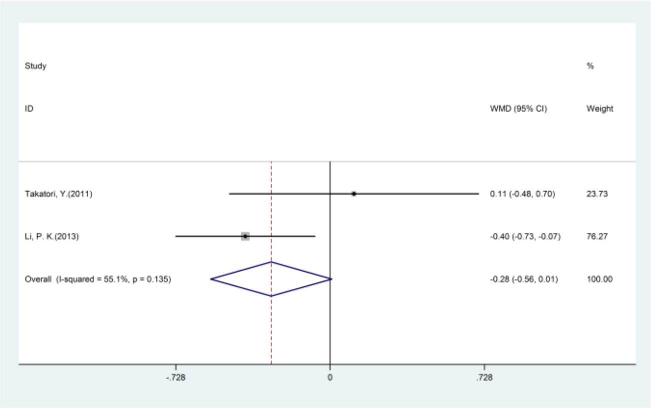

Figure 34. Forest plot of comparison (in subgroups of diabetes, <6 month): ICO vs GLU: Outcome: LDL-C (mmol/L).

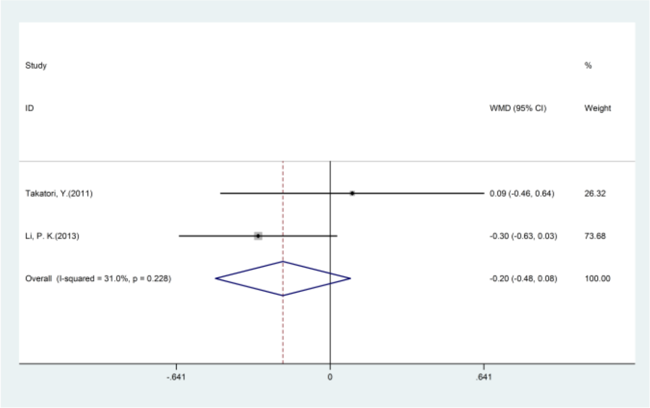

Figure 35. Forest plot of comparison (in subgroups of diabetes, ≥6 month): ICO vs GLU: Outcome: LDL-C (mmol/L).
